# Anellovirus constraint from 2 to 6 months postpartum followed by betatorquevirus and gammatorquevirus dominance in serum and milk

**DOI:** 10.1128/jvi.00846-25

**Published:** 2025-07-31

**Authors:** Anne L. Timmerman, Antonia L. M. Schönert, Martin Deijs, Jacqueline van Rijswijk, Marit J. van Gils, Johannes B. van Goudoever, Britt J. van Keulen, Lia van der Hoek

**Affiliations:** 1 Laboratory of Experimental Virology, Department of Medical Microbiology and Infection Prevention, Amsterdam UMC, University of Amsterdam 1234, Amsterdam, the Netherlands; 2 Amsterdam Institute for Infection and Immunity, Amsterdam, the Netherlands; 3 Department of Pediatrics, Amsterdam UMC, University of Amsterdam 1234, Amsterdam, the Netherlands; 4 Amsterdam Reproduction & Development Research Institute, Amsterdam, the Netherlands; Cornell University Baker Institute for Animal Health, Ithaca, New York, USA

**Keywords:** colonization, gammatorquevirus, betatorquevirus, alphatorquevirus, human milk, compartmentalization, anellovirus, TTV

## Abstract

Anelloviruses are ubiquitous, diverse viruses in the human virome not causing disease. In adults, the anellovirus composition (anellome) is dominated by alphatorqueviruses. However, infants seem to acquire their first anellovirus infections beginning with beta*-* and gammatorquevirus, soon followed by alphatorquevirus. Transmission via human milk is suggested as a potential transmission route. Consequently, we hypothesize that milk may contain predominantly beta- and gammatorqueviruses. That would also imply that compartmentalization between milk and serum takes place as the anellome in adults is dominated by alphatorqueviruses. We therefore examined anellovirus distribution in paired serum and milk samples of lactating mothers in the first 15 months after delivery (*n* = 30). Unexpectedly, genus-specific qPCRs revealed that the majority of serum and milk were negative for anelloviruses 2–6 months postpartum (4 serum and 1 milk positives out of 13), whereas positive from 6 months postpartum onwards (11 serum and 8 milk positives out of 16). Importantly, a dominance of beta- and gammatorquevirus in both serum and milk from 6 months postpartum was found. We also found significantly higher anellovirus loads in serum compared to milk, with no obvious difference in anellovirus genera presence between compartments. Anellovirus variant analysis using Oxford Nanopore sequencing revealed that 31 out of 45 variants were shared with the corresponding serum of the mother. We conclude that anellovirus compartmentalization between the circulation and the mammary glands could not be detected.

**IMPORTANCE:**

Anelloviruses are omnipresent in the human population, yet their transmission routes, especially in early life, remain unclear. This study focused on compartmentalization between blood and the mammary gland to investigate the likelihood of virus transmission via milk. We show that serum and milk samples generally share anellovirus genera and lineages. Interestingly, serum as well as milk samples collected between 2 and 6 months postpartum tested mainly negative for anelloviruses, but those received thereafter were dominated by beta- and gammatorquevirus, reflecting the initial anellovirus colonizers observed in children.

## INTRODUCTION

Infecting the vast majority of the human population (1), anelloviruses belong to the most prevalent viruses colonizing human hosts, occupying over 70% of a healthy human’s virome (2). The lack of a disease association has prompted researchers to categorize the virus as commensal (reviewed by Kaczorowska and van der Hoek [3]). A range of studies has identified anelloviruses in a plethora of sample types, including blood, semen, vaginal fluids, feces, nasal secretions, bone marrow, and saliva (4–11). Children are generally born negative, yet first anellovirus infection occurs already within the first months after birth, followed by lifelong viral persistence (12–14).

With their size ranging from 2.0 to 3.9 kb, anelloviruses are small, single-stranded circular DNA viruses of negative sense that contain overlapping open reading frames and an untranslated region (reviewed by Kaczorowska and van der Hoek [3]). Differing in genome size, the most well-known and commonly found genera in humans are classified as: *Alphatorquevirus*, *Betatorquevirus,* and *Gammatorquevirus* (15). Rarely, three other genera, *Hetorquevirus, Memtorquevirus,* and *Samektorquevirus* can also be detected (16). Most individuals are colonized not by one, but by several lineages of more than one genus, making co-infections a common phenomenon (14, 17, 18). Taken together, the collection of anelloviruses circulating in a single person is referred to as the anellome, which is highly personalized and stable over time (14).

The exact mode of transmission of viruses to newborns is not known; however, oral transmission is a likely route (19). Anelloviruses have been detected in saliva and human milk (13, 20–22). The anellome of infants younger than 12 months is made up of predominantly betatorqueviruses and gammatorqueviruses, whereas alphatorqueviruses contribute less to the anellome (13, 23, 24). In contrast, the adult blood virome contains mainly alphatorqueviruses and less frequently beta- and gammatorqueviruses (14, 25–27). Interestingly, we recently found that in milk from lactating mothers, alphatorqueviruses were only rarely detectable and the most prevalent genera were beta- and gammatorqueviruses (13). This lack of alphatorquevirus dominance in milk is thus far unexplained. It is possible that the viral distribution in milk differs from the viral distribution in the blood of the mother, a phenomenon referred to as compartmentalization (28). Compartmentalization of anelloviruses between the mammary gland as one compartment and the blood circulation as the other compartment is an area that has remained unexplored.

Compartmentalization between two tissues has been observed for some viruses, such as human immunodeficiency virus 1 (HIV-1) (29), hepatitis C virus (HCV) (30), and more recently for severe acute respiratory syndrome coronavirus 2 (31). Compartmentalization of viral lineages can influence transmission by filtering the lineages that are transmitted (28, 29, 32, 33). The alveoli located in the mammary glands, in which milk is produced, are encompassed by a network of capillaries to ensure proper exchange of oxygen and nutrients but also allow viruses to enter the compartment (34). Viruses, such as HIV-1, cytomegalovirus (CMV), or Zika virus, are found in milk and can either be transported into the milk via immune cells or transcytosed through mammary epithelial cells.

The aim of this study is to investigate whether compartmentalization of anelloviruses occurs between blood and the mammary glands of lactating women. We investigated the prevalence of anelloviruses in 30 mothers that donated paired serum and milk samples. Compartmentalization may explain the early colonization of beta- and gammatorquevirus in children.

## RESULTS

### Compartmentalization of anelloviruses between blood and mammary gland

**Fig 1:**
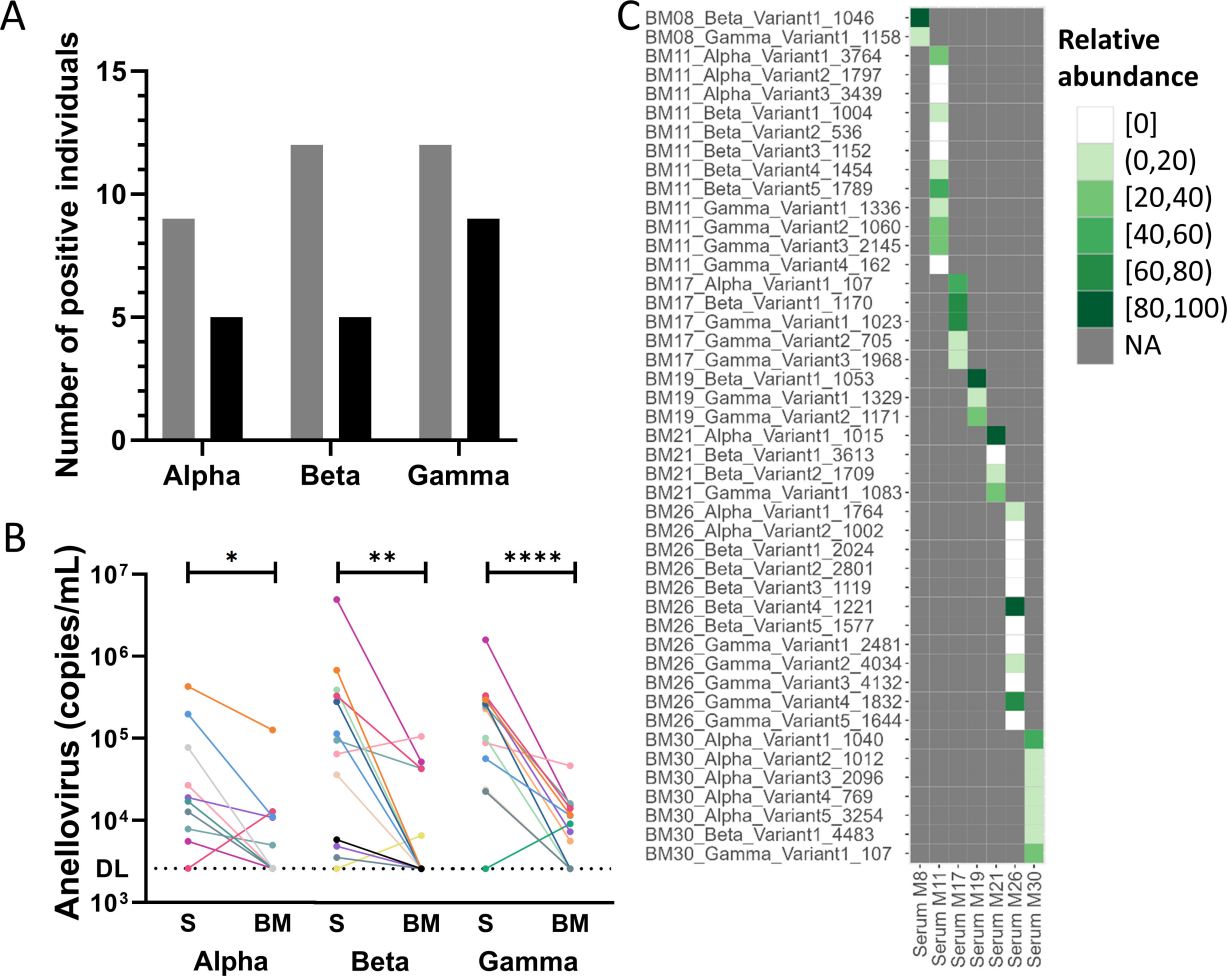
Anellovirus prevalence and load between paired serum and milk samples. (**A**) Number of individuals positive for alpha-, beta-, and gammatorquevirus. Gray and black represent the anellovirus prevalence in serum and milk, respectively. (**B**) Anellovirus load categorized per anellovirus genus and serum (S) and milk (BM) origin. Each color represents the anellovirus loads detected in serum and milk of a single mother, and the lines connect the samples of the same individual. (**C**) Heatmap of milk reference anellovirus variants found in the serum of the corresponding mothers. Reference variants from milk are shown on the *Y*-axis. Intensity of the green color represents viral read abundance. *P < 0.05, **P < 0.01, and ****P < 0.0001.

To investigate potential compartmentalization between the blood and mammary gland, alpha-, beta-, and gammatorquevirus concentrations were measured using qPCRs in 30 paired human serum and milk samples of lactating women collected within the first 15 months after delivery (Table S1). In milk, 10 samples contained detectable anellovirus loads. Two out of 10 positive samples were positive for all three genera. Double genera infections were also detected, as three milk samples contained alpha- and gammatorqueviruses, and two samples beta- and gammatorqueviruses. Three milk samples were positive for one genus only; one sample for betatorquevirus and two samples were only gammatorquevirus positive. Gammatorqueviruses were most abundantly found, as a total of nine samples tested positive (30%), compared to five positive betatorquevirus (17%), and five alphatorquevirus positives (17%) (Fig. 1A).

A higher frequency of positive anellovirus qPCR results was found in serum compared to milk. In serum, 15 mothers showed detectable anellovirus loads (Table S1). Of those, most mothers (*n* = 7) were positive for all three genera in serum. Four serum samples showed both beta- and gammatorquevirus detection. Only a few samples showed single alpha*-* (*n* = 2), beta*-* (*n* = 1), or gammatorquevirus (*n* = 1) detection. Betatorqueviruses were thus detected in 12 of the samples (40%), 12 tested positive for gammatorqueviruses (40%), and alphatorquevirus could be observed in 9 serum samples (30%) (Fig. 1A). The differences in genus prevalence between serum and milk were not significantly different (*P* value = 0.662 [alphatorquevirus], *P* value = 0.137 [betatorquevirus], *P* value = 1 [gammatorquevirus]) (Table S2).

Next, we compared genus prevalence within each individual between serum and milk. For alphatorquevirus, four mothers were positive in both compartments, while five mothers showed only detectable alphatorquevirus levels in serum and one mother was alphatorquevirus positive in milk (Fig. 1B). Out of 12 mothers with detectable betatorquevirus in serum, four mothers were betatorquevirus positive in both compartments, while eight mothers only presented with detectable viral loads in serum. Only one mother was betatorquevirus positive in milk, while remaining negative in serum. For gammatorquevirus, eight mothers tested positive in both compartments, four were gammatorquevirus positive only in the serum, and one mother showed gammatorquevirus only in the milk.

As viral load can serve as an indicator for independent replication, which is considered a principal property inherent to compartmentalized strains (29), it can provide insight on whether the anelloviruses in milk are subjected to independent viral replication. We compared the viral loads of all genera within a compartment and between paired serum and milk samples. When comparing the loads between genera within the same compartment, the genus-specific anellovirus concentrations did not differ within serum (*P* value = 0.1232; Table S3) or milk (*P* value = 0.6936; Table S3). Nevertheless, when comparing genus loads between serum and milk, significantly lower anellovirus loads were found in milk samples compared to serum for all genera (*P*-value < 0.05 [alphatorquevirus], *P* value < 0.01 [betatorquevirus], *P* value < 0.0001 [gammatorquevirus]; Fig. 1B). A Pearson *r* correlation test between the two compartments was performed, which indeed showed a significant correlation for alphatorquevirus (Pearson *r* = 0.6891, *P* value < 0.0001; Table S4), betatorquevirus (Pearson *r* = 0.5680, *P* value < 0.01), and gammatorquevirus (Pearson *r* = 0.6910, *P* value < 0.0001), indicating that compartmentalization does not occur.

Due to the direct correlation of anellovirus genus load between compartments, and the significantly lower milk anellovirus concentrations compared to serum concentrations, it could be that viral leakage or non-selective viral transfer from blood into milk takes place. We next investigated whether serum load could be considered a suitable predictor for milk prevalence. Using a simple logistic regression, a significant association between the anellovirus load in serum and the anellovirus prevalence of the corresponding genus in milk was indeed found (alphatorquevirus: *P* value < 0.01, OR = 8.492 [95% CI = 1.836–75.46]; betatorquevirus: *P* value < 0.05, OR = 3.446 [95% CI = 1.307–12.32]; gammatorquevirus: *P* value < 0.0001, OR = 9.597 [95% CI = 2.9–58.98]; Table S5).

To inspect the anellome in more detail, on the level of individual lineage distribution, genus-specific PCR amplified Oxford Nanopore sequencing was performed on a selection of the paired samples. Selection of the samples was based on anellovirus presence in both the serum as well as in the paired milk sample, and sufficient anellovirus concentration in the milk sample (>10^4^ copies/mL) for at least one of the genera. This resulted in sequencing of the anellovirus lineages in serum and milk samples of seven mothers (M8, M11, M17, M19, M21, M26, and M30) (Table S6). All milk variants were assembled with 95% nucleotide identity to select unique anellovirus reference variants in each mother. In total, 45 unique anellovirus variants were found: 12 alphatorqueviruses, 16 betatorqueviruses, and 17 gammatorqueviruses (Table S7). Nanopore sequenced reads originating from the corresponding serum samples were subsequently aligned to the references from milk (per mother) and quantified as a hit at a read depth >5 reads and 95% identity. Out of 45 unique anellovirus variants found in milk, a majority of 31 were also detected in serum. In four out of seven mothers (M8, M17, M19, and M30), all variants represented in the milk anellome were detectable in the paired serum sample. For M11, out of 12 anellovirus variants, 5 were absent in the serum: 2 alphatorqueviruses*,* 2 betatorqueviruses*,* and 1 gammatorquevirus variant. The milk anellome of mother 21 contains four anellovirus variants, of which only one, a betatorquevirus variant*,* was not detectable in the serum. The only outlier was mother 26, with a majority of variants solely found in milk (8 out of 12) (Fig. 1C). An analysis of unique variants from serum and their presence or absence in milk was not performed, because this was beyond the scope of the study.

### Time dependency of anelloviruses postpartum

**Fig 2:**
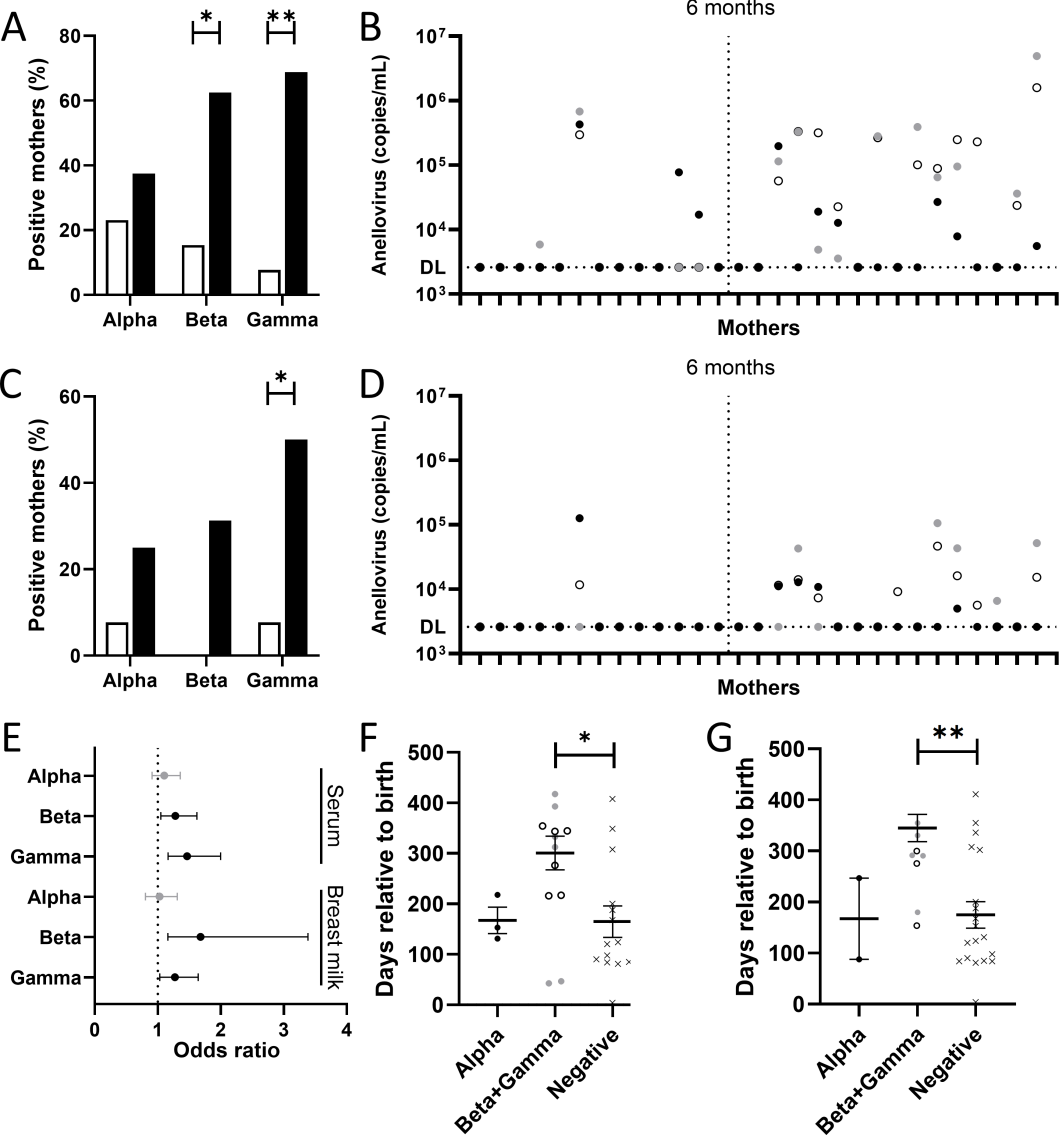
Anellovirus prevalence and concentration in mothers postpartum. Number of (**A**) serum and (**C**) milk positive samples for alpha-, beta-, and gammatorquevirus up to 6 months (white bars) and after 6 months (black bars) postpartum. Anellovirus concentrations in (**B**) serum and (**D**) milk of women following childbirth. Serum samples are shown on the *X*-axis in ascending order of lactation duration. The vertical line corresponds to 6 months postpartum. (**E**) Odds ratio and confidence intervals (CIs) of the association between days relative to birth and anellovirus incidence in serum and milk, using simple logistic regression. Black error bars represent significant 95% CIs. (**F**) Number of days relative to birth, categorized by alphatorquevirus dominance, beta- and gammatorquevirus dominance, or anellovirus negative in their serum or (**G**) milk sample. Black circles represent alphatorquevirus, white circles betatorquevirus, and gray circles symbols gammatorquevirus. Black crosses refer to anellovirus negative samples. *P < 0.05 and **P < 0.01.

We next investigated the anellovirus composition over time in the serum-milk paired samples. We performed an ascending lactation duration-based categorization of participants, subsequently comparing differences in anelloviruses. Of the 30 tested individuals, 13 mothers were sampled within the first 6 months after delivery. Of note, 12 of these 13 were collected between 2.5 and 6 months after delivery. Sixteen individuals donated their samples between 6 and 15 months of lactation duration. The time of donation after delivery was unknown in one sample and consequently left out of this part of our research. Of the 13 women who donated material before 6 months postpartum, only 4 mothers (31%) showed detectable anelloviruses in serum (Fig. 2A and B). In comparison, 11 out of 16 mothers (69%) who donated after 6 months postpartum presented with detectable anelloviruses in serum. This difference was statistically significant for beta- and gammatorquevirus between serum collected after 6 months compared to the first 6 months postpartum (*P* value < 0.05 [betatorquevirus], *P* value < 0.01 [gammatorquevirus] (Table S9). This effect was not found for alphatorquevirus (*P* value = 0.909).

The anellovirus prevalence over time was also found to be different in milk, with only one positive anellovirus sample in the first 6 months (8%), and 8 out of 16 women positive for anelloviruses in milk after the 6-month mark (50%) (Fig. 2C and D). Especially gammatorquevirus infections show a significantly higher prevalence 6 months after birth compared to the preceding 6-month period (*P* value < 0.05) (Table S9). This difference was not statistically significantly different for alpha- and betatorquevirus in milk (*P* value = 0.686 [alphatorquevirus], *P* value = 0.095 [betatorquevirus]).

The unexpected time dependency of lactation duration and anellovirus detection was further analyzed using simple logistic regression. In serum, a significant association was found between time and betatorquevirus (*P* value < 0.05, OR = 1.279 [95% CI = 1.049–1.623]) as well as gammatorquevirus (*P* value < 0.001, OR = 1.467 [95% CI = 1.163–1.997]), but not for alphatorquevirus infections (*P* value = 0.316, OR = 1.104 [95% CI = 0.9104–1.359]) (Table S10; Fig. 2E). In milk, a similar pattern can be observed, with a significant association between time and betatorquevirus (*P* value < 0.01, OR = 1.679 [95% CI = 1.160–3.386]) and gammatorquevirus detection (*P* value < 0.05, OR = 1.273 [95% CI = 1.033–1.643]), but a non-significant association between time and alphatorquevirus detection (*P* value = 0.819, OR = 1.028 [95% CI = 0.8061–1.311]). To further investigate the time-dependent dynamics influencing the anellovirus genera load, we categorized the serum samples based on the genus dominance, classifying them as dominated by alpha-, beta-/gammatorquevirus, or as negative. Next, we compared the days between birth and donation of the material between the three abovementioned classifications. We observed a significantly longer interval of days between birth and donation in mothers with beta-/gammatorquevirus dominance compared to the negative group in serum (*P* value < 0.05) and milk (*P* value < 0.01) (Fig. 2F and G).

### Cytokine production postpartum

**Fig 3:**
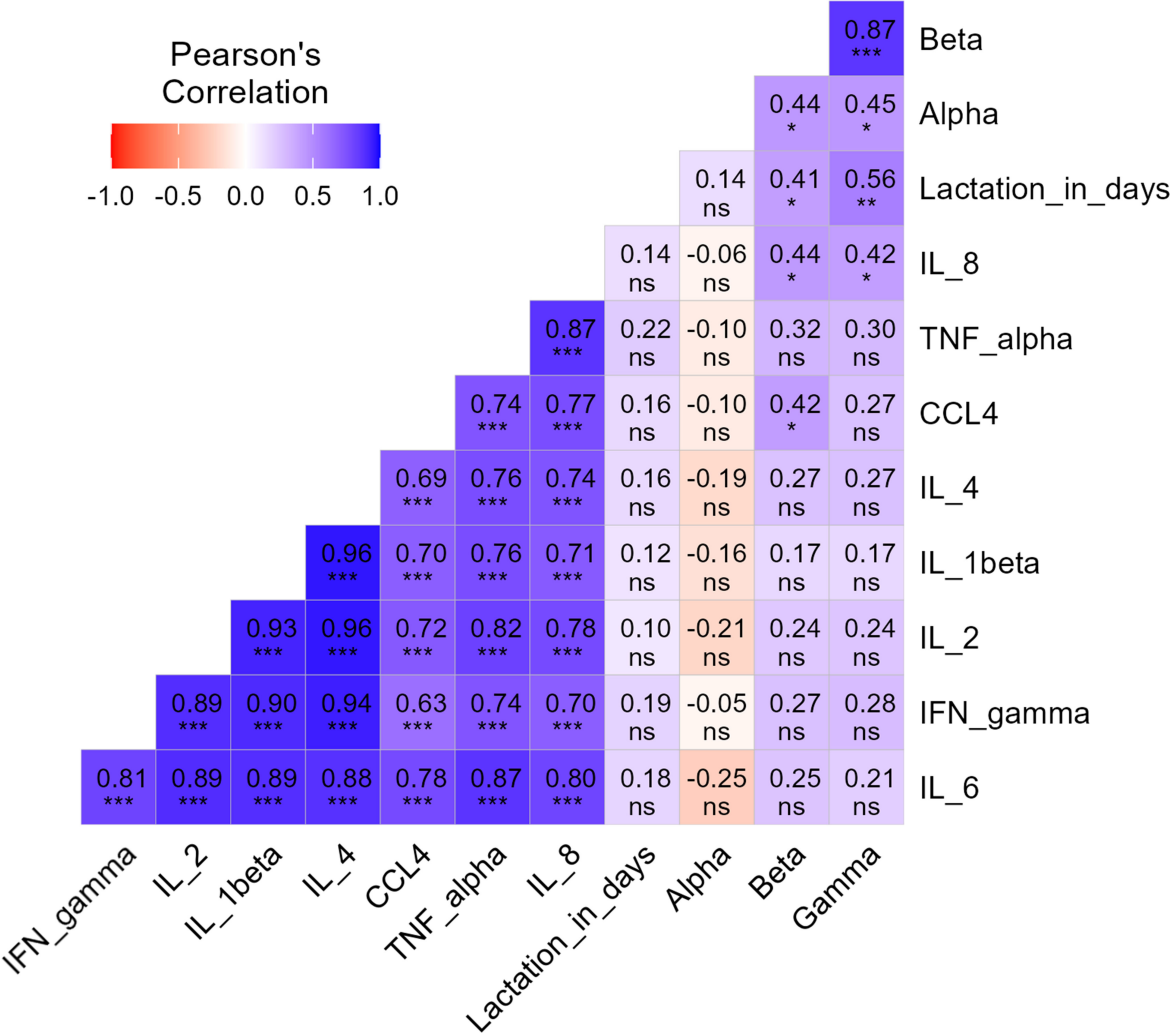
Correlation plots of cytokines in serum versus detection of alpha-, beta-, and gammatorqueviruses and time post-delivery. Correlation plots between cytokine levels (IFN-γ, IL-2, IL-1β, IL-4, CCL4, TNF-α, and IL-8), anellovirus concentration (alpha-, beta-, and gammatorquevirus) and lactation in days. Color and numbers indicate correlation coefficient and the asterisk indicates the significance. *P < 0.05, **P < 0.01, and ***P < 0.001.

Given the dominance of beta- and gammatorquevirus between 6 and 15 months after delivery, we considered that postpartum immune states may influence a mother’s anellome. After giving birth, there are substantial immune state fluctuations (35, 36). We therefore measured cytokine levels in the serum with known time of donation after delivery (*n* = 29). Cytokine concentrations were quantified using a Luminex bead-based immunoassay and cytokines playing a role in postpartum immunity: TNF-α, IL-6, IL-8, IL-1β, IFN-γ, CCL4, IL-4, and IL-2. As expected, a negative correlation coefficient, although not significant, was observed between all the measured cytokine levels and alphatorquevirus. Unexpectedly, significant positive correlations were found between IL-8 and betatorquevirus (*r* = 0.44, *P* value < 0.05), IL-8 and gammatorquevirus (*r* = 0.42, *P* value < 0.05), and between CCL-4 and betatorquevirus (*r* = 0.42, *P* value < 0.05) (Fig. 3).

## DISCUSSION

In this study, we investigated the anellome distribution postpartum. Strong signs of compartmentalization between the blood and mammary glands of the breast were not found on the level of genus distribution between blood and the mammary glands. Instead, the obtained data showed the distributions as an equilibrated state, with both prevalence and viral loads of the genera following a shared pattern throughout the two different compartments. Nanopore sequencing showed that the majority of variants present in milk were shared with the corresponding serum of the mother, indicating the absence of compartmentalization.

The composition of the blood anellome in lactating mothers diverged from the expected distribution in non-lactating adults. Instead of the higher alphatorquevirus prevalence and dominance normally found in adults (14, 25–27), beta- and gammatorqueviruses outcompeted alphatorqueviruses in the mothers. It could be that female gender plays a role in the uncommon genus distribution found in our study. Most of the studies researching anelloviruses in men and women found no significant difference between gender and alphatorquevirus dominance in blood (27, 37–40). Two studies did, however, find a significant difference between men and women, showing a higher alphatorquevirus prevalence for men (40, 41). Unfortunately, these studies provided no information on possible pregnancies, and it is therefore not known if the materials from some of these women were collected soon after delivery. There are two other studies with indirect indications that time since delivery may have an impact on a woman’s anellome. A study done by Haloschan et al. (42) found higher alphatorquevirus concentrations in adult men compared to women, but only in the age group between 20 and 30 years (42). Another study in the Romanian population found a higher number of gammatorquevirus positives in women compared to men, but only in the age group between 31 and 40 years (27). The age groups of women with unexpectedly low alphatorqueviruses coincide with the age during which most women experience pregnancy. Whether just the difference in sex or delivery/pregnancy influences the distributions of the anellovirus genera deserves future attention, especially since alphatorquevirus concentrations are used as markers for proper immunosuppressive therapy after solid organ transplantations (43).

Not only did we find beta- and gammatorqueviruses dominating the anellomes of the lactating mothers, but this prevalence was highest 6 months after birth. It might be that behavioral changes account for the rises in anellovirus concentrations, occurring 6 months postpartum, such as the mother returning to work with the infant starting day care. Also, changes in feeding patterns of the child occur around 6 months of age; however, a study performed by Beller et al. (44) showed little influence of type of feeding on anelloviruses. They analyzed the gut virome in eight longitudinally sampled infants and found no differences in the accumulation of unique anellovirus contigs when children changed their feeding pattern from milk to formula or solid food (44).

Considering that children seem first colonized by beta- and gammatorqueviruses (13), it could be possible that, instead of mother-to-child transmission, a child-to-mother transmission takes place. Interestingly, a study investigating alpha- and betatorquevirus load during pregnancy discovered a generally higher concentration of both genera in blood and saliva in women with at least one prior birth, compared to women who had never given birth before (45). In addition, an increased anellovirus prevalence was found in women who had given birth compared to women who had never given birth (27). One study showed that children by the age of 3 months can harbor anelloviruses that do not originate from the mother (46), and it could thus be that anelloviruses from infants are indeed, with time, transmitted to the mother. We hypothesize that infants may obtain their first anelloviruses from the other parent or other infants (e.g., siblings or day-care “friends”) (44). Transmission from the infant oral cavity towards the mammary duct, known as retrograde flow, has been observed for some microbes, such as oral bacteria (47). Still, this route seems unlikely for anelloviruses due to the lower anellovirus load we found in milk compared to serum. Oral-oral or fecal-oral transmission is a more likely route for child-to-mother transmission.

As delivery approaches, the heightened immune activation near the end of the third trimester is driven by increased leukocyte migration towards the uterus, increased endocrine hormones, and elevated pro-inflammatory cytokine production (35, 36). We anticipated that the abrupt increase in inflammation at delivery may induce an immunological shock, providing a potential explanation for the cluster of anellovirus-negative samples that were collected between 2 and 6 months postpartum. Spontaneous viral clearance within the first year postpartum has been reported for other viruses, including HCV and human papillomavirus (48, 49). Although changes of the immune system postpartum are not fully characterized, restoration of the immune system towards a pre-pregnant state can take up to 1 year after delivery (50). The postpartum period is characterized by increased helper T-cells and cytotoxic T-cells from 1 to 4 months, increased suppressor T-cells from months 4 to 10, and increased B-cells between 7 and 10 months postpartum (50). In addition, certain cytokines are known to fluctuate after delivery, including TNF-α (51–53), IL-6 (51–53), IL-8 (52, 53), IL-1β (52), IFN-γ (51, 54), CCL4 (55, 56), IL-4 (54), and IL-2 (51, 54). We found a significant positive correlation between higher IL-8 and CCL-4 and more frequent detection of beta- and gammatorquevirus. CCL4, also known as macrophage inflammatory protein (MIP) 1β, is an effector molecule that is secreted by activated leukocytes and muscle cells (57). CCL4 acts as a chemoattractant for several immune cells, including natural killer cells and monocytes. IL-8 is also named chemokine (C-X-C motif) ligand 8 (CXCL8) and is expressed by epithelial cells and macrophages to induce migration of neutrophilic granulocytes towards inflamed tissue (57, 58). Immune cells may play a double role in anellovirus persistence: on the one hand, they may combat the virus, while on the other hand, they could facilitate viral replication. Since anelloviruses are also detected in leukocytes (59–63), it may be likely that the increase of immune cells postpartum leads to higher anellovirus concentration in blood. A negative, although not significant, correlation was indeed found between cytokine levels and alphatorquevirus loads in our study. Less expected was the abovementioned positive correlation for beta- and gammatorquevirus, and CCL4 and IL-8. This highlights the discrepant behavior of beta-/gammatorquevirus on the one hand and alphatorquevirus on the other, suggesting that beta- and gammatorqueviruses probably infect a different subset of cells compared to alphatorqueviruses.

A limitation of this study is that almost all samples—30 out of 31—were collected between 2.5 and 15 months after delivery. Only one sample was collected shortly, within 1 week, after delivery. In an earlier study in human milk, we found anelloviruses in 11 out of 34 milk samples donated within the first 2 weeks after delivery, of which alphatorqueviruses showed the highest concentrations compared to the other genera (Table S1 of Kaczorowska et al. [13]). More research is needed on anelloviruses close to the delivery date. A second limitation is the unknown efficiency of anellovirus DNA isolation when guanidine thiocyanate and silica binding are the basis of nucleic acid isolation. Varying performance with milk as starting material has been described for different isolation techniques and the Qiagen-based assay, also using the guanidine isothiocyanate, performed less than other isolation methods (64). A third limitation is the absence of serum samples collected during pregnancy. We therefore do not know if the personal anellome was already dominated by beta- and gammatorqueviruses prior to delivery.

In conclusion, a significantly higher beta- and gammatorquevirus prevalence was found in both serum and milk collected 6–15 months after partition compared to samples collected at earlier breastfeeding moments. We encourage future studies with longitudinally collected samples from mothers during their pregnancy until 1 year after delivery, ideally in combination with samples of the newborns, to determine the full details of anellovirus colonization.

## MATERIALS AND METHODS

### Clinical samples

Paired serum-milk samples were part of the study “COVID MILK-POWER MILK,”, conducted between October 2020 and February 2021, by the Amsterdam UMC. For the current study, 30 paired serum and milk samples were included from mothers who (i) gave birth for the first time, and (ii) donated in October 2020 (Table S1). Participants were requested to collect 10–30 mL of milk in a sterile container (SteriFeed) from the first feeding moment in the morning of their study appointment, where maternal blood was also collected. Additionally, participants were asked to fill out a questionnaire asking about lactation duration, age, and date of delivery. The milk and serum samples were stored at −80°C.

### Nucleic acid isolation

110 μL of the thawed serum sample was centrifuged for 10 min at 5,000 × *g*, and 100 µL of supernatant was transferred into a new tube. The sample was subsequently treated with a TURBO DNase (ThermoFisher, Karlsruhe, Germany, AM2238) to remove background DNA, including microbial and genomic DNA that could interfere with the Illumina sequencing. Nucleic acids within intact virus particles were extracted according to the BOOM isolation method (65). Nucleic acids were eluted using 65 µL Baker water (VWR, 4218). Milk samples were not centrifuged due to the viscosity of the milk, and 100 µL of the milk sample was directly used for DNase treatment and nucleic acid isolation by the BOOM method.

### Genus-specific qPCRs

Genus-specific qPCRs were performed to detect alpha-, beta-, and gammatorqueviruses, as previously described (14). No strict genus-specific conserved stretches can be identified for gammatorqueviruses, and the primers used for gammatorquevirus detection can therefore also amplify some betatorqueviruses. For the purpose of simplicity, though, the qPCR is referred to as a gammatorquevirus qPCR. The qPCR reaction mixture contained 6.25 µL of 2× Qiagen RotorGene Probe Master-mix (Qiagen GmbH, Hilden, Germany, 204374), 0.25 µL of forward primer (20 µM), 0.25 µL of reverse primer (20 µM), 0.25 µL of Probe (10 µM), 3 µL of Baker water, and 2.5 µL of nucleic acid sample. Cycling profile: 3 min at 95°C, followed by 40 cycles of 3 s at 95°C and 10 s at 60°C, with a final elongation step of 3 min at 72°C. No template containing controls were included in each run to monitor potential cross-contamination. Dilutions of a positive plasmid control were used as a standard curve and provided the basis for the calculations of the viral DNA concentrations of the samples. The cut-off was set at 10 copies/reaction.

### Nanopore NGS libraries preparation

Nucleic acids were PCR amplified, targeting the non-coding region (NCR), using the genus-specific anellovirus primers, as previously described (14). The reaction contained 5 µL isolated nucleic acids, 25 µL DreamTaq Green PCR Master Mix (Thermo Fisher Scientific, CATK1081), 0.5 µL forward and 0.5 µL reverse primer (both 20  µM), and 19 µL H_2_O. The PCR started at 95°C for 5 min, followed by 45 cycles of 95°C for 30 s, 55°C for 30 s, and 72°C for 1 min each, combined with a final elongation step at 72°C for 10 min. PCR products were purified by adding AMPure XP beads (Agencourt AMPure XP, Beckman Coulter, Woerden, the Netherlands) at a ratio of 1:1.8 (product: AMPure XP beads), followed by incubation for 10 min at room temperature. Beads were washed two times using 70% ethanol and eluted in 20 µL of Baker water. PCR products were sequenced at Plasmidsaurus using Oxford Nanopore sequencing Premium PCR (South San Francisco, CA, USA).

### Anellovirus prevalence using Nanopore sequencing

Raw sequence reads (.fastq files) were trimmed using seqkit in miniconda3 (seq function) using the following parameters: minimal length (-m), maximal length (-M), alphatorquevirus -m 95M 216, betatorquevirus -m 136M 176, and gammatorquevirus -m 142M 220. Reads were trimmed using the corresponding primer sequences, listed previously (14), using Dorado (Oxford Nanopore, http://nanoporetech.com, Version 0.8.2) and converted into .bam files. The .bam files were converted into .fastq files using Samtools (version 1.9) using the fastq parameter (66). The trimmed milk sequences were further analyzed by aligning the reads with primer sequences removed at 95% identity using CodonCode Aligner (version 10.0.2). A reference required a read depth above five reads. The references were further analyzed using the BLASTn alignment to the NCBI nucleotide database (67). For serum samples, unprocessed reads (.fastq files) were aligned to the milk reference variants at 95% identity using CodonCode Aligner. A detected variant was considered a hit if a read depth of above five reads was found.

### Cytokine detection using Luminex Assay

Cytokine levels in serum were determined using the magnetic bead-based Human Luminex Discovery Assay (LXSAHM-08, Bio-Techne Corporation, Minneapolis, MN, USA), according to the manufacturer’s protocol. A custom-ordered bead assay was used, which included detection of the following cytokines known to fluctuate during pregnancy and postpartum: TNF-α (51–53), IL-6 (51–53), IL-8 (52, 53), IL-1β (52), IFN-γ (51, 54), CCL4 (55, 56), IL-4 (54), and IL-2 (51, 54). Readout of the plates was performed on the Magpix system (Luminex, Bio-Techne Corporation, Minneapolis, MN, USA).

### Statistical analysis

A McNemar’s test was used to compare genus prevalence between paired serum and milk samples. A mixed-effects model was used to compare the genus viral load within milk or serum. A paired *t*-test was used to compare viral loads between paired serum and milk samples. Additionally, the correlation between anellovirus load in serum and milk was inspected using the Pearson correlation test. The association between the anellovirus load in serum and anellovirus prevalence in milk was tested using simple logistic regression. Anellovirus prevalence was compared between each time category using Fisher’s exact test. Simple logistic regression was used to test the association between time and anellovirus prevalence. One-way ANOVA test with Tukey’s multiple comparisons was used to compare days between birth across anellovirus dominance groups. Spearman correlation was used to compare cytokine levels, anellovirus loads, and time of lactation. All figures were created using Graphpad Prism (version 10.2.0), except for the heatmaps and correlation plot, which were generated using RStudio (version 4.4.1). Significance was defined as *P* ≤ 0.05 for all statistical tests.

## Data Availability

Additional information and resource requests should be directed to the lead contact, Lia van der Hoek (c.m.vanderhoek@amsterdamumc.nl). Host-removed reads and reference variants from the milk using Oxford Nanopore sequencing can be found on GitHub (https://github.com/AnneTimmerman/Compartmentalisation).

## SUPPLEMENTAL MATERIAL

Supplemental tables jvi.00846-25-s0001.xlsx Tables S1 to S10.

ASM does not own the copyrights to Supplemental Material that may be linked to, or accessed through, an article. The authors have granted ASM a non-exclusive, world-wide license to publish the Supplemental Material files. Please contact the corresponding author directly for reuse.
